# Gut microbiota profiling with differential tolerance against the reduced dietary fibre level in rabbit

**DOI:** 10.1038/s41598-018-36534-6

**Published:** 2019-01-22

**Authors:** Shi-Yi Chen, Feilong Deng, Xianbo Jia, Hanzhong Liu, Gong-Wei Zhang, Song-Jia Lai

**Affiliations:** 1 Farm Animal Genetic Resources Exploration and Innovation Key Laboratory of Sichuan Province, Sichuan Agricultural University, Chengdu, China; 2 Sichuan Academy of Grassland Sciences, Chengdu, China; 3 College of Animal Science, Southwest University, Rongchang, China

## Abstract

Dietary fibre is well acknowledged to be critical in maintaining the gut homeostasis in human and other monogastric animals. As a small monogastric herbivorous animal, rabbit is much sensitive to the reduced intake of dietary fibre and more interestingly shows individual difference in clinical tolerance. In the present study, we fed rabbits with fibre-deficiency diet for two weeks and successfully distinguished the individual tolerances according to clinical signs and gastrointestinal gross lesions. A total of 40 treatments were classified into three groups of the full health (N = 10), moderate intestinal disorder (N = 11) and severe intestinal disorder (N = 19). Together with three controls, 43 individuals were subjected to gut microbiota profiling by 16S rRNA gene sequencing. It was revealed that the Firmicutes/Bacteroidetes ratio steadily decreased from 1.74 in healthy group to 1.03 in severe group. However, the healthy individuals that showed complete tolerance still remained a comparable Firmicutes/Bacteroidetes ratio with controls. Notably, the class Alphaproteobacteria was found to be higher abundance in healthy group than controls and other treatment groups. The results would improve our understanding of the relationship among dietary fibre, gut microbiota and host health.

## Introduction

Dietary fibre, which is defined as carbohydrate polymers with ten or more monomeric units and indigestible in small intestine^1^, has been widely acknowledged to have many health benefits in human and other monogastric animals^2,3^. Over the past decades our dietary habits, especially in western countries, have dramatically shifted toward a lower intake of fibre as well as excessive consumption of the refined fats and sugars, which would be a key factor to drive incidences of obesity, diabetes and inflammatory bowel diseases worldwide^4^. In the livestock production, fibre is also an important dietary component for maintaining normal physiological function in the digestive tract for monogastric animals^5–7^.

The physiological effects of dietary fibre are associated with gut microbiota composition and metabolic profiles, gastrointestinal motility and immunity, and other systemic consequences^8^. Because mammalian genomes fail to encode active enzyme for the structural carbohydrates, dietary fibre is absolutely degraded via microbial fermentation within either hindgut of monogastric herbivores and omnivores or rumen for ruminants^9,10^. The major fermented products of dietary fibre are short-chain fatty acids mainly consisting of acetate, propionate and butyrate^11^, which play important roles for modulating host immune responses and could also serve as energy sources of metabolism for both host and gut microorganisms^12–15^. Therefore the dietary fibre content and sources would have important roles in maintaining the gut homeostasis that is a mutually beneficial balance between gut microbiota and host, whereas both gut microbiota and host genetic background could conversely affect the degradation of dietary fibre^16–18^.

In addition to health implication of the long-term dietary pattern, changes in gut microbiota can be immediately observed even after short-term consumption of the altered diets, such as conversion between animal-based and plant-based diets^19–21^. Rabbit (*Oryctolagus cuniculus*) belongs to the family Leporidae and has been widely used as animal model in biomedical researches^22^. As a small monogastric herbivorous animal, rabbit is much sensitive to the dietary fibre content that low dietary fibre level could quickly and obviously induce intestinal disorders^23^. According to our field observation and other reports^24^, there are individual differences in clinical tolerance against the fibre-deficiency diet. A few candidate genes, such as Toll-like receptor 4 (*TLR4*) and myeloid differentiating factor 88 (*MyD88*), had been identified in our former studies to be associated with intestinal disorders of rabbit^25,26^. However, it still remains largely unknown about the link between gut microbiota and differential tolerance against the reduced intake of dietary fibre. In the present study, we fed rabbits with fibre-deficiency diet and successfully distinguished individual tolerance, for which the gut microbiota was analyzed comprehensively using high-throughput sequencing approach.

## Materials and Methods

### Ethics statement

The study design was approved and all methods were performed in accordance with guidelines of Institutional Animal Care and Use Committee in College of Animal Science and Technology, Sichuan Agricultural University.

### Experimental design

**Figure 1:**
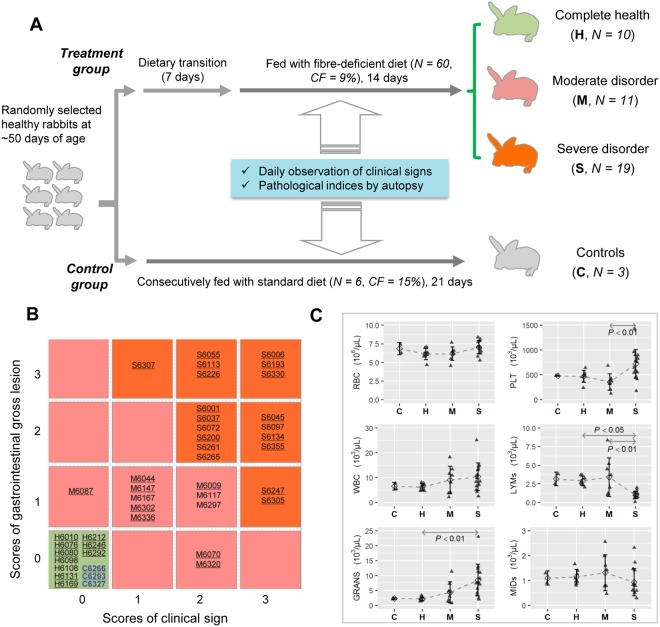
Experimental design and individual classification. (**A**) A total of 66 rabbits are initially collected and randomly classified into the treatment and control groups. At the end of experimental period, 40 treatments and three controls were finally included for gut microbiota profiling. (**B**) Matrix plot shows our classification of the 43 samples according to scores of clinical signs and gastrointestinal gross lesions. Background colors of boxes indicate different groups and all Sample IDs are also prefixed by the abbreviation of groups. The samples that have haematological results are underlined. (**C**) The six haematological indices (Mean ± SD) are compared among four groups with pairwise *t* test of significant difference and Bonferroni adjustment of multiple comparisons.

We initially collected a total of 66 healthy New Zealand White rabbits at ~50 days of age, which were randomly classified into the treatment (N = 60) and control (N = 6) groups, respectively. Rabbits in treatment group were fed with fibre-deficient diet for two weeks after a 7-day dietary transition, whereas control group was consecutively fed with standard diet (Fig. 1A). The designed contrast between fibre-deficient and standard diets is the fibre content with crude fibre of 9% *vs*. 15%, acid-detergent fibre of 13% *vs*. 21% and neutral-detergent fibre of 29% *vs*. 36% (Supplementary Table S1). Throughout experimental period at the room temperatures of 26 ± 3 °C, all rabbits were fed the pelleted diet *ad libitum* without any antimicrobial exposure and housed in individual cages under our standard management protocol.

Two indices in relation to intestinal health were taken into consideration for evaluating individual responses to the reduced dietary fibre level, including (i) each rabbit was carefully observed twice a day for precisely recording clinical signs of intestinal disorders throughout experimental period; (ii) all rabbits were slaughtered and subjected to an autopsy for checking gastrointestinal gross lesions at the end of experimental period. All rabbits were classified according to the two indices that were independently scored by three experienced colleagues and only those individuals that could be unambiguously classified (N = 40) were included for the gut microbiota profiling. Together with three randomly selected controls, a total of 43 rabbits were finally subjected to the high-throughput sequencing.

### Scoring of clinical signs and gastrointestinal gross lesions

We precisely observed the clinical sign in relation to intestinal disorder for each rabbit, including the disturbance of food intake, diarrhoea, constipation (caecal impaction) and presence of mucus in excreta. Based on daily records during the whole experimental period for these clinical signs, every rabbit was empirically and averagely scored with four levels. By macroscopic observation for each part of gastrointestinal tract (stomach, duodenum, jejunum, ileum, caecum and colon) at necropsy, symptoms and lesions were clearly described, including the watery content, dilatation, impaction, congestion and mucus. Similarly, four levels were used to subjectively score the gross lesions of gastrointestinal tract. The description of scoring standard was shown in Table 1, and the higher score was associated with more severe intestinal disorder. It is required that the three experts must arrive at a consensus for each individual. Apart from the gastrointestinal tract, all rabbits didn’t show obvious gross lesion at other sites.

**Table 1:** Scoring standard for the clinical signs and gastrointestinal gross lesions.

Clinical signs		Gross lesions	
Score	Description	Score	Description
0	No any clinical sign	0	No any gross lesion
1	Disturbance of food intake or unshaped stools	1	Abnormal liquid content or gas observed in stomach and in limited segments of small intestine
2	Light diarrhea or abdominal swelling	2	Abnormal liquid content or gas widely observed in small and large intestines
3	Acute diarrhoea, constipation or presence of mucus in excreta	3	Abnormal liquid contents or gas observed in whole digestive tract and presence of mucus in colon

### Hematological analyses

The whole blood samples were collected by vacuum-based blood collector with K_2_·EDTA. Hematological analyses were immediately performed using an automatic hematological analyzer of Medonic CA620 (Boule Medical, Stockholm, Sweden). Finally, we measured six haematological indices, including the total red blood cell count (RBC), platelet count (PLT), white blood cell count (WBC), lymphocyte count (LYMs), neutrophil count (GRANS) and mid cell population count (MIDs).

### 16S rRNA gene sequencing

To avoid the sampling bias, the entire caecal contents were first blended sufficiently and then collected for each rabbit. The total DNA was extracted from caecal content (about 200 mg) using QIAamp DNA Stool Mini Kit (Qiagen, Shanghai, China). After checking quality of the extracted DNA by NanoVue Plus (GE Healthcare, Piscataway, USA), the V3 region of bacterial 16S rRNA gene was amplified using HOTSTAR Taq Plus Master Mix Kit (Qiagen, Shanghai, China) and the universal primers (338 F: 5′-ACT CCT ACG GGA GGC AGC AG-3′ and 533 R: 5′-TTA CCG CGG CTG CTG GCA C-3′)^27^. The PCR condition involved an initial denaturation step at 95 °C for 4 min and 20 cycles of 95 °C for 1 min, 56 °C for 45 sec, and 72 °C for 1 min, and followed by an extension step at 72 °C for 7 min using a Bio-Rad CFX96 thermal cycler (Bio-Rad, Hercules, USA).

Each sample was independently amplified in triplicate, which were pooled and then purified using QIAquick PCR Purification Kit (Qiagen, Shanghai, China). Amplicons with both a total amount of ≥3 μg and OD260/280 ratio ≥1.8 were used to produce sequencing libraries using Illumina DNA Sample Preparation Kit (Illumina, San Diego, USA) according to manufacturer’s instructions. Finally, the libraries were sequenced on Illumina HiSeq™ 2000 platform for generating the 150 bp paired-end reads.

### Bioinformatic analysis

For the raw reads of 16S rRNA gene sequencing, we unconditionally cut the first base and trimmed adaptor sequences using Cutadapt tool^28^. Paired-end reads were merged to produce tags using USEARCH tool with the minimum overlapping length of 25 bp and minimum total length of 100 bp; and all the produced tags were further filtered according to the expected error probability of 0.25^29^. After both forward and reverse primers were removed, we finally obtained the clean tags.

Subsequently, we employed UPARSE-OTU algorithm for clustering operational taxonomic units (OTUs) according to the recommended steps and similarity threshold of 0.03^30^, in which all singletons were also discarded. Based on the representative tag, every OTU was taxonomically annotated using RDP naïve Bayesian classifier with confidence value of 80%^31^. For each sample, the community richness (Chao1 and ACE) and diversity (Shannon, Simpson and Invsimpson) at OTU rank were calculated using VEGAN R package^32^. Using STAMP tool^33^, all samples were clustered by principal component analysis (PCA) and statistical deduction of differentially abundant features among the four groups was conducted by one-way analysis of variance (ANOVA) and Tukey-Kramer post hoc test. The Benjamini-Hochberg procedure was used for controlling the false positive rate in multiple comparisons. Additionally, the unclassified sequences are only included for denoting the relative proportion of feature, which is calculated relative to the total number of sequences being assigned to its parent category at phylum or grandparent category at other levels.

Raw files of gut microbiota sequencing reads are available at NCBI Sequence Read Archive (SRA) under Bioproject PRJNA393739.

## Results

### Differentiation of individual tolerance against fibre-deficiency diet

Among the 60 rabbits initially included in the treatment group, a total of 40 individuals could be unambiguously scored for both clinical signs and gastrointestinal gross lesions (Supplementary Table S2), and all of them were finally classified into three groups (Fig. 1B), including the full health (Healthy, N = 10), moderate intestinal disorder (Moderate, N = 11), and severe intestinal disorder (Severe, N = 19). Therefore, it is roughly estimated that about 25% and 50% of individuals show the complete tolerance and develop into severe intestinal disorder upon the reduced dietary fibre level, respectively. Together with the three controls consecutively fed with standard diet (Control, N = 3), a total of 43 samples were subjected to the following analyses.

Because we finally failed in preparing the qualified blood samples, the six haematological indices were successfully collected only for 33 rabbits (Supplementary Table S2). The inter-group comparisons revealed that there were no significant differences for three indices of RBC, WBC and MIDs (Fig. 1C). However, rabbits from severe group were associated with the significantly increased PLT, LYMs and GRANS (*p* < 0.05). All six haematological indices didn’t show significant differences among the control, healthy and moderate groups. The results would also support our classification of rabbits to indicate differential health states.

### 16S rRNA gene sequencing and OTU picking

**Figure 2:**
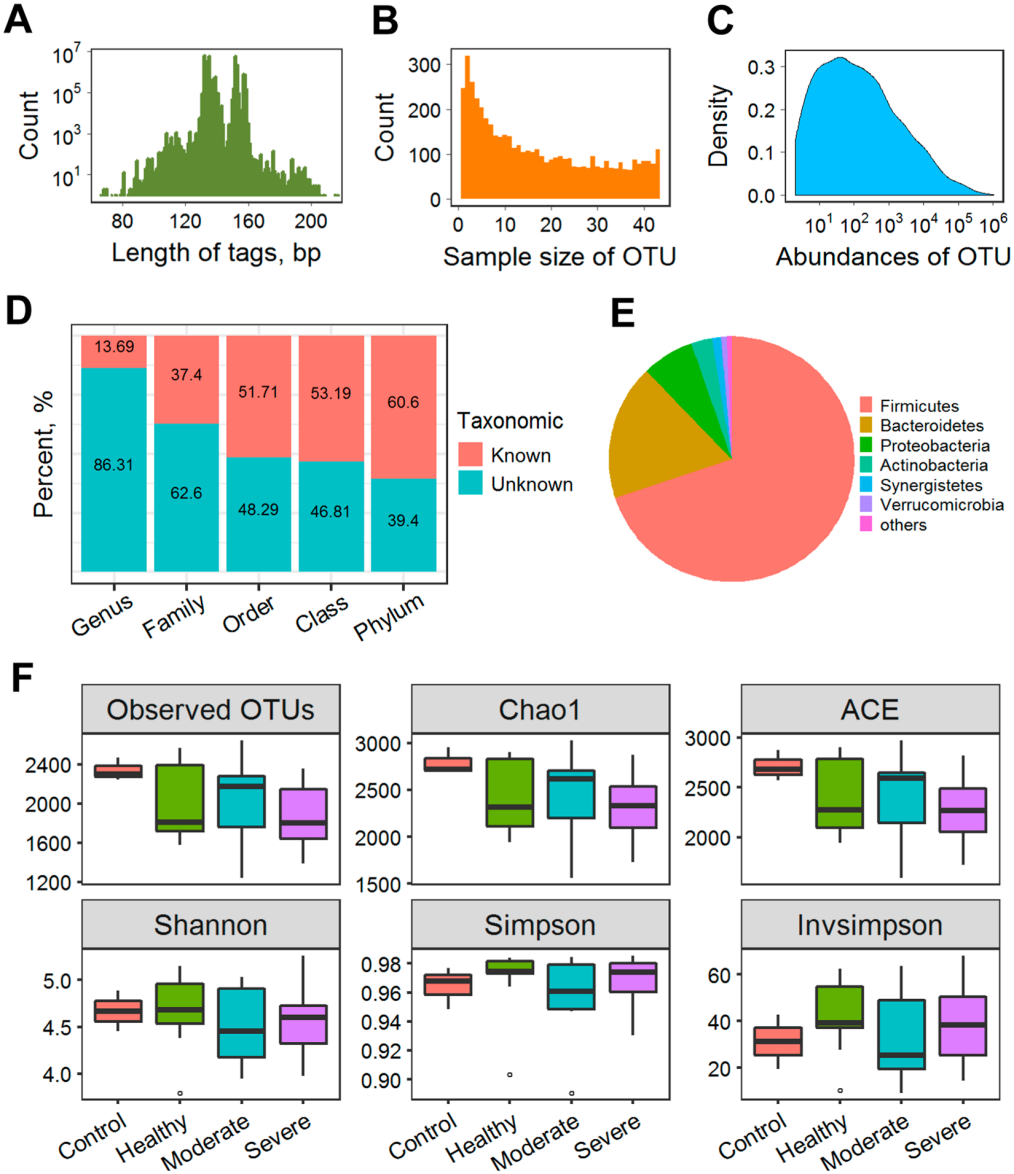
OTU picking, taxonomic assignment and microbial diversity. Length distribution of clean tags is first shown (**A**). The constructed OTUs show differential sample size that is defined as the number of samples sharing an OTU (**B**) and absolute abundance of tag (**C**). All OTUs are counted according to taxonomic assignments at different levels (**D**), among which the phylum-level composition is demonstrated (**E**). Finally, six indices in relation to richness and diversity of microbial community are schematically compared among the four groups with box and whiskers plot (**F**).

A total of 81.65 million raw paired-end reads of 16S rRNA gene sequencing were generated among all individuals, which produced 59.13 million raw tags (Supplementary Table S2). After quality filtering, we got 27.76 million clean tags with the most predominant lengths at both 135 bp and 152 bp (Fig. 2A). After removing 47,806 unique tags that had been computationally deduced to be chimeric sequences, all tags were subjected to OTU picking and herein produced 4,931 OTUs with the skewed frequency distribution for both sample size and absolute abundance (Fig. 2B,C). More than 60% of the constructed OTUs were taxonomically assigned to 14 phyla, whereas only a small fraction (13.69%) was successfully annotated at the genus level (Fig. 2D). Among the taxonomically annotated OTUs, phyla Firmicutes and Bacteroidetes were absolutely predominated with the observed frequencies of 69.8% and 17.9%, respectively (Fig. 2E).

### Compositional differences of gut microbiota

Both community richness and diversity were calculated and compared among the four groups according to the established OTU profile (Fig. 2F). Beside control group that has only three samples, the three treatment groups showed comparable species richness and inter-individual variation in terms of the observed OTUs, Chao1 and ACE. For the community diversity, there was also no obvious difference among the four groups. On the whole, Shannon index was less variable among groups than Simpson and Invsimpson indices.

**Figure 3:**
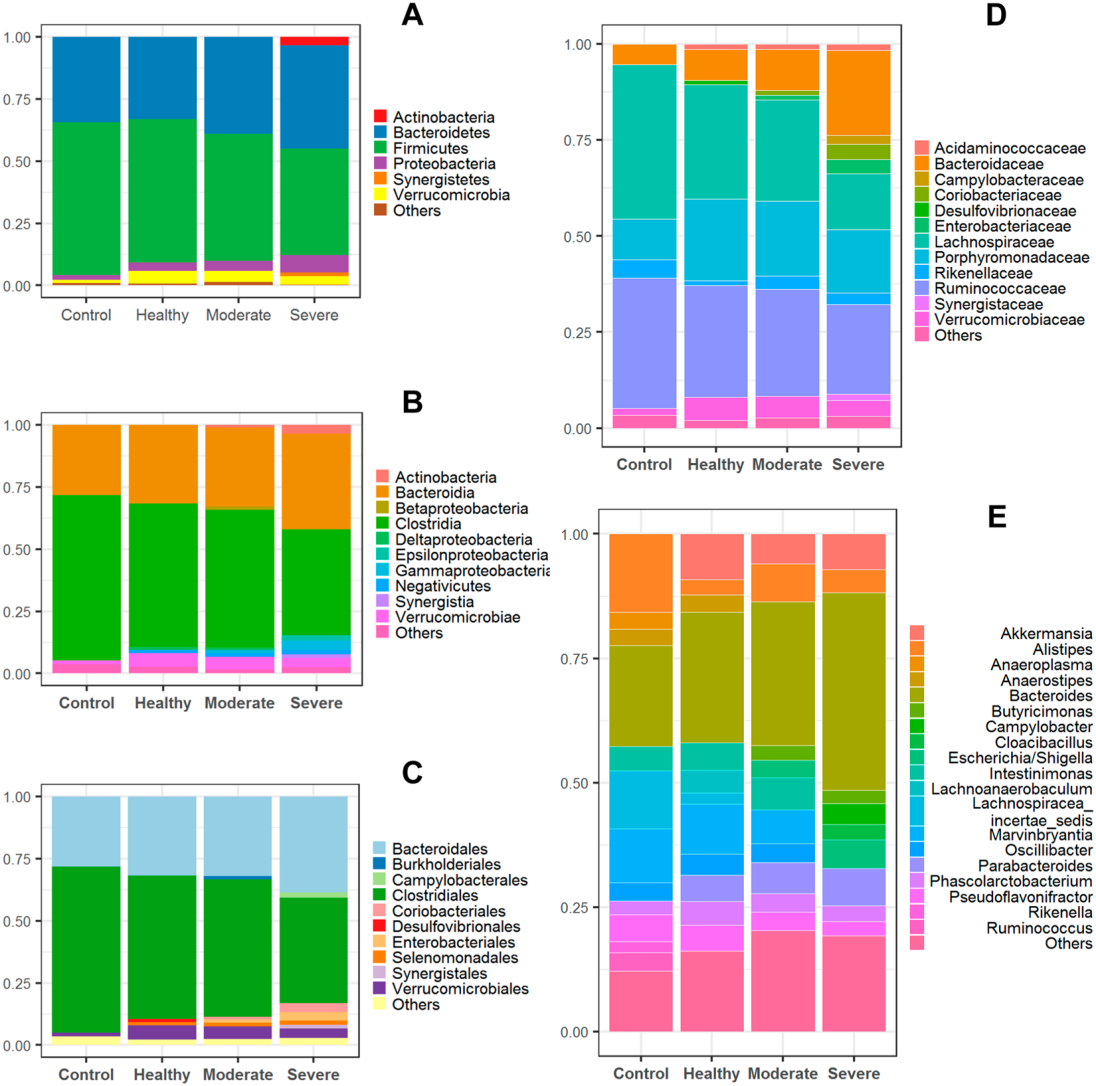
Composition of the annotated taxonomies. The OTUs are taxonomically annotated at Phylum (**A**), Class (**B**), Order (**C**), Family (**D**) and Genus (**E**) levels, respectively. The relative proportion of abundance is shown as mean among all individuals in each group. The taxonomies with the relative abundance lower than 0.02 at Genus level and 0.01 at other levels are merged into the catalog of “Others”.

For each group, we also investigated the relative proportion of the taxonomically annotated OTUs (Fig. 3), from which inter-group differences could be directly observed at various levels. The Firmicutes/Bacteroidetes ratio steadily decreased from control (1.79), healthy (1.74), moderate (1.32) to severe (1.03) groups at the phylum level (Fig. 3A); and the Actinobacteria in the severe group (3.4%) was much more abundant than others. At order level, Desulfovibrionales (1.2%) was almost exclusively present in healthy group (Fig. 3C). On the whole, we observed the higher proportion in both healthy and moderate groups for the phylum Verrucomicrobia, class Verrucomicrobiae, order Verrucomicrobiales and family Verrucomicrobiaceae than that in the control and severe groups.

### Statistical analysis

**Figure 4:**
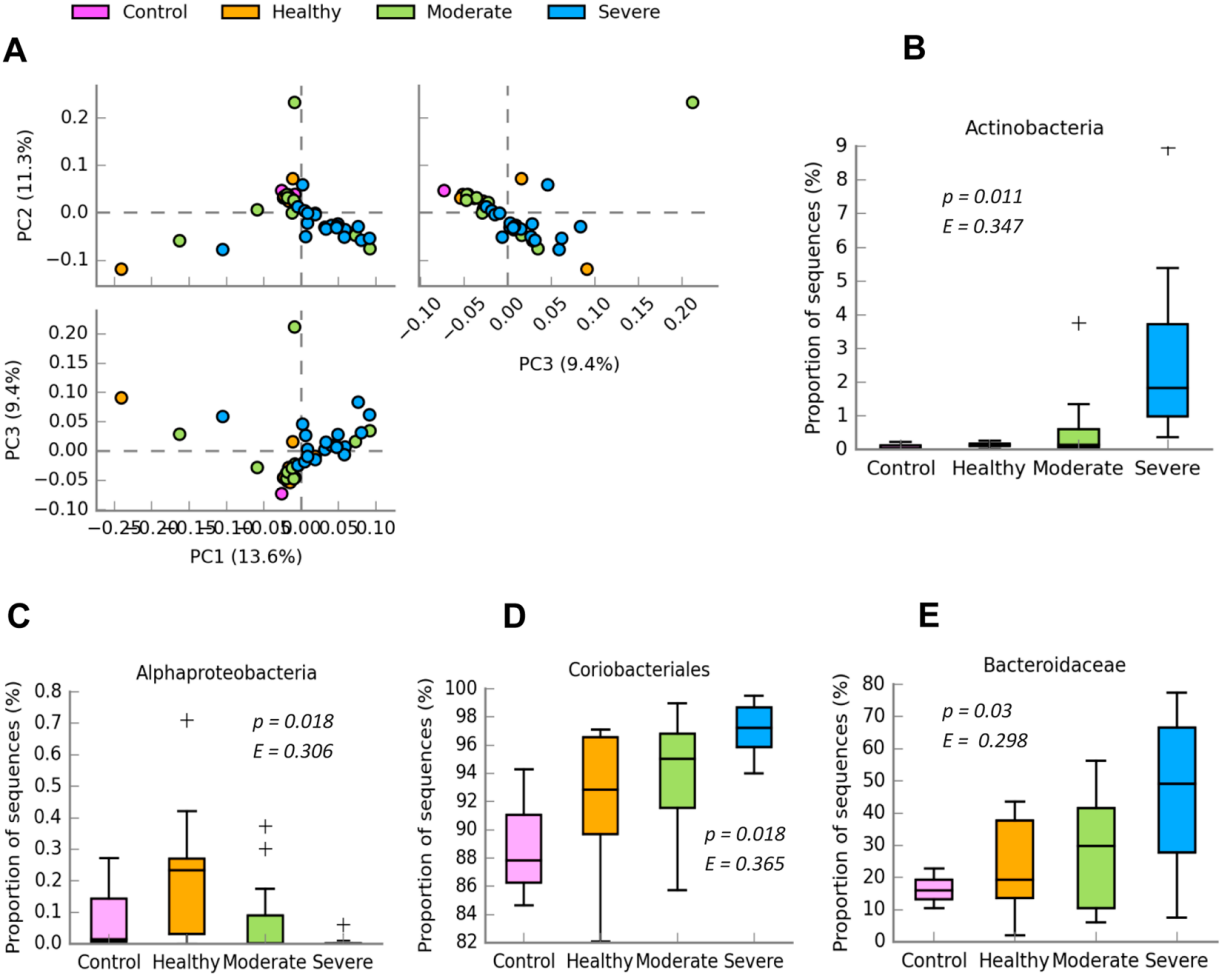
Sample clustering and differential abundance analysis. Sample clustering is revealed by PCA method (**A**). Box plots show the significant differences in abundance for the phylum *Actinobacteria* (**B**), class *Alphaproteobacteria* (**C**), order *Coriobacteriales* (**D**) and family *Bacteroidaceae* (**E**), respectively.

**Figure 5:**
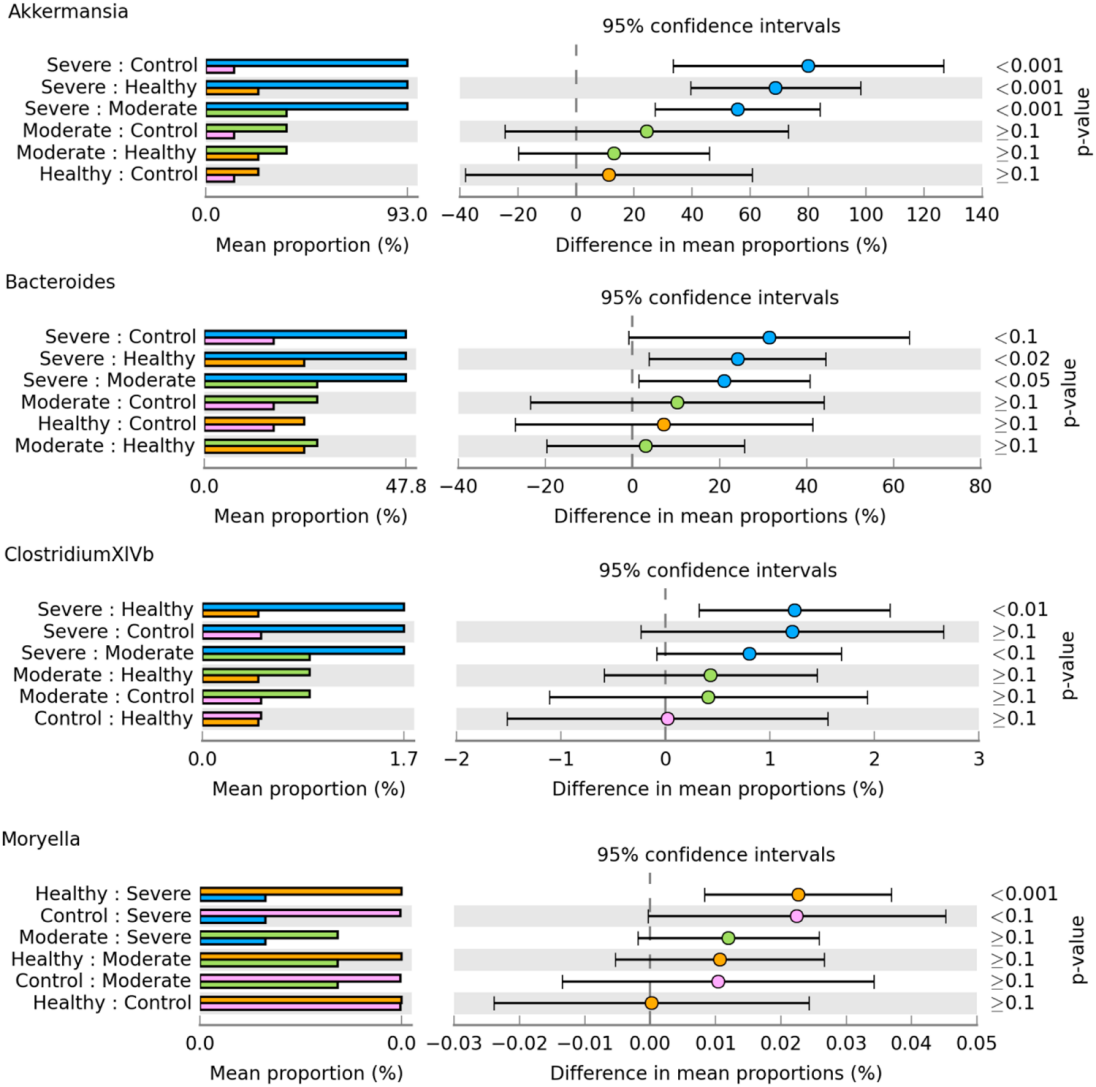
Post host test for the differential abundance. Bar plots (Left) show the average proportion in each group, for which pair-wise comparisons are further shown as 95% confidence intervals (Right).

We first employed PCA method for clustering all samples based on the OTU profile (Fig. 4A), in which more than 34% of variances were explained by the three principal components. In contrast to moderate and severe groups, the individuals in healthy group were less clustered. Among the 14 taxonomically annotated phyla, only phylum Actinobacteria was revealed to be significant differential abundance among the four groups (Fig. 4B). Furthermore, we observed significant differences in abundance for the classes of Alphaproteobacteria (Fig. 4C) and Actinobacteria, order Coriobacteriales (Fig. 4D), and families of Bacteroidaceae (Fig. 4E), Coriobacteriaceae and Lachnospiraceae. At genus level, four genera of Akkermansia, Bacteroides, ClostridiumXlVb and Moryella were significantly differentially abundant among the four groups, for which the post hoc tests were shown in Fig. 5. The healthy group showed a higher abundance for genus Moryella than severe group (*p* < 0.001).

## Discussion

Although dietary fibre has little nutritional merit per se in human, its health benefits for preventing many chronic diseases have been well acknowledged^2,3^. Therefore, adequate intake of dietary fibre was recommended by American Dietetic Association with 25–38 g/day for adults^34^. However, the fundamental mechanism underlying health benefits of dietary fibre has remained largely unknown. Unlike human and other rodents, rabbit is much sensitive to the dietary fibre content and obviously shows individual difference in clinical tolerance against fibre-deficiency diet^24,25^. In this study, we fed rabbits with fibre-deficiency diet over a relatively short period of time and successfully established the animal model of intestinal disorders induced by deficiency of dietary fibre. This feeding experiment clearly distinguished individual outcomes in response to the reduced dietary fibre level and estimated that ~25% of individuals show complete tolerance, whereas almost half the individuals also develop into server intestinal disorders. These individual differences would be helpful for understanding the impact of dietary fibre on intestinal health.

The beneficial and detrimental impacts of gut microbiota on host health have been widely revealed^35^. In human and other mammals, the gut microbiota is typically dominated by two bacterial phyla of Firmicutes and Bacteroidetes and the Firmicutes/Bacteroidetes ratio is also apt to be influenced by dietary pattern^36^. By investigating fecal microbiota, it was revealed that children from rural Africa with long-term consumption of fibre-rich diet have higher abundance of Bacteroidetes and less Firmicutes than that of European children^19^. In a follow-up trial, healthy adults with a short-term supplementation of dietary fibre similarly showed the decreased Firmicutes/Bacteroidetes ratio^16^. In contrast, the decreased Firmicutes/Bacteroidetes ratio was also observed in rabbits fed with fibre-deficiency diet in the present study. However, the healthy individuals that showed complete tolerance against the reduced dietary fibre level still remained a comparable Firmicutes/Bacteroidetes ratio with controls. Our results would provide additional evidence for understanding relationship among dietary fibre, gut microbiota composition and host heath.

In addition to the altered Firmicutes/Bacteroidetes ratio, phylum Actinobacteria was also revealed to be highly abundant in severe group but almost completely absent from the healthy individuals and controls. Therefore, the increased abundance of Actinobacteria is likely a consequence of the developed severe intestinal disorders. Additionally, we observed that healthy rabbits with complete tolerance against the reduced dietary fibre level had the highest abundance of class Alphaproteobacteria in comparison with both controls and other treatment groups. This result would likely suggest a potential association of Alphaproteobacteria with individual tolerance against the reduced dietary fibre level. We also found that a higher abundance of Alphaproteobacteria was reported in patients with Crohn’s disease^37,38^. However, the functional implications of class Alphaproteobacteria for regulating individual response to deficiency of dietary fibre should be investigated in the future.

## Conclusion

In the present study, we fed rabbits with the fibre-deficiency diet and distinguished individual response to such diet intervention. Using the high-throughput sequencing approach, we comprehensively investigated gut microbiota composition and found that the Firmicutes/Bacteroidetes ratio would be associated with differential tolerance.

## Electronic supplementary material

Supplementary Table S1

Supplementary Table S2
