# Supplementary material for: Gut microbiota profiling with differential tolerance against the reduced dietary fibre level in rabbit
Source: Sci Rep. 2019 Jan 22;9:288. doi: 10.1038/s41598-018-36534-6 (PMC6342909; doi:10.1038/s41598-018-36534-6)
Supplement: Supplementary file 1 — Supplementary Table S1 [file 41598_2018_36534_MOESM1_ESM.docx]

**Supplementary Table S1** Ingredients and nutritive values of the experimental diets.

| **Compositions** | **Fibre-deficient diet** | **Standard diet** |
| --- | --- | --- |
| **Ingredients** (%) | | |
| corn | 25.8 | 15.8 |
| wheat bran | 19.8 | 17.0 |
| wheat-middling | 11.0 | 5.0 |
| corn bran | 10.8 | 5.0 |
| rice bran | — | 3.0 |
| Soya bean meal | 14.9 | 9.0 |
| rapeseed cake | 5.0 | 4.0 |
| alfalfa meal | 9.0 | 38.0 |
| Calcium carbonate | 1.0 | 0.9 |
| Calcium Hydrogen Phosphate | 1.5 | 1.1 |
| Sodium chloride | 0.3 | 0.3 |
| L-lysine | 0.125 | 0.125 |
| DL-methionine | 0.075 | 0.075 |
| Vitamine/mineral premix | 0.7 | 0.7 |
| **Nutritive values (air dry basis)** | | |
| Crude Protein (%) | 14.26 | 14.65 |
| Digestive Energy (MJ/kg) | 11.50 | 11.06 |
| Crude fiber (CF, %) | 8.89 | 15.16 |
| Acid-detergent fibre (ADF, %) | 13.38 | 21.19 |
| Neutral-detergent fibre (NDF, %) | 29.21 | 35.71 |
